# Prevalence of oral mucosal lesions among smokers based on gender: a bicentric cross-sectional study in Egypt

**DOI:** 10.1186/s12903-026-10040-y

**Published:** 2026-11-03

**Authors:** Dalia Ghalwash, Fat’heya M. Zahran

**Affiliations:** 1 Oral Medicine and Periodontology, Faculty of Dentistry, The British University in Egypt, El Sherouk City, Cairo, 11837 Egypt; 2 Oral Medicine and Periodontology, Faculty of Dentistry, Cairo University, Cairo, Egypt

**Keywords:** Tobacco use, Oral diseases, Sex factors, Tongue coating, Leukoedema, Tobacco-related oral conditions, Smoker's melanosis, Leukoplakia

## Abstract

**Background:**

Smoking is a major risk factor for developing various types of oral mucosal lesions (OMLs), including benign and potentially malignant lesions. However, scarce data is available concerning gender differences regarding the prevalence of oral mucosal lesions in smokers worldwide. Accordingly, this study assesses the prevalence and types of OMLs among male smokers compared to female smokers in an Egyptian population.

**Methods:**

This hospital-based cross-sectional study included 300 male and 300 female smokers. All participants underwent a thorough case history review and a comprehensive clinical examination.

**Results:**

The overall prevalence of oral lesions was significantly higher in males (75%) than in females (52%). Increased tongue coating, black hairy tongue, leukoedema, and smoker’s palate were found in males more than in females, with a statistically significant difference. Leukoplakia, a potentially malignant disorder, showed an odds ratio in males more than twice that of females. In contrast, males were found to have a significantly lower risk of developing smokers’ melanosis than females.

**Conclusion:**

Both males and females manifest the same types of oral mucosal lesions in relation to smoking; however, males have an overall significantly higher prevalence of such lesions, with a higher odds ratio to develop certain types, including leukoplakia. Hence, gender-specific awareness programs are needed, aided by targeted male smokers’ education and oral screening by their dentists and health care providers.

**Supplementary Information:**

The online version contains supplementary material available at https://doi.org/10.1186/s12903-026-10040-y.

## Introduction

Tobacco is a powerful addictive material that changes a person’s mental, emotional, and social demeanors [1]. Tobacco smoking is a major health problem, representing the second leading risk factor for global disease and economic burden, leading to more than six million deaths worldwide, which may escalate to 8 million deaths every year by 2030 [2]. Therefore, tobacco cessation is one of the important goals for the prevention and control of chronic diseases, disability, and mortality [3].

Various types of tobacco forms are produced and marketed, which can be smoked in different forms available in the market, providing many varieties [4]. Smoking challenges the oral cavity with the resultant heat, in addition to about 7,000 chemicals and free radicals that can be absorbed into the oral mucosa. Nicotine, carbon monoxide, acetaldehyde, nitric oxides, and radium are considered the most important components. Polycyclic aromatic hydrocarbons, nitrosamines, and aromatic amines are the most significant carcinogens [5]. Smoking can thus affect the oral mucosa, which acts as a protective physical barrier against trauma, pathogens, and carcinogenic irritants [6]. It also results in oxidative stress, DNA and cellular damage, and affects oral keratinocytes’ plasma membrane integrity, increasing epithelial thickness and keratinization [7]. Epithelial cells might even undergo dysplastic changes, leading to the development of potentially malignant disorders and oral cancer, according to the frequency and duration of smoking [7, 8].

Smoking can have a negative impact on a person’s oral health-related quality of life as it is a major risk factor for developing various types of oral mucosal lesions (OMLs), some of which are benign as, increased tongue coating, black hairy tongue, smoker’s keratosis, smoker’s melanosis, and smoker’s palate [4]. In contrast, others may have the potential to undergo malignant transformation, such as leukoplakia, erythroplakia, and lichen planus, which are termed oral potentially malignant disorders (OPMDs). It is also a major risk factor for the development of oral squamous cell carcinoma [9, 10]. In addition, smoking represents an important systemic modifying factor for the progression of periodontal diseases [11]. The increase in such tobacco-associated lesions is also observed in young populations, not just in adults [12].

The investigation of OML prevalence in specific population groups is mandatory to understand its extent and characteristics. Still, it is also essential for the improvement of oral health promotion and prevention programs for specific genders or age groups, as recommended by the World Health Organization (WHO) [13]. However, scarce data are available concerning gender differences regarding the prevalence and types of oral mucosal lesions in smokers worldwide.

Tobacco smoking is alarmingly prevalent among low- and middle-income countries, with 80% of the annual tobacco-related mortality expected to occur in these countries by 2030 [2]. Therefore, it is imperative to understand the implications of smoking on oral health. According to the WHO global report on trends in the prevalence of tobacco use 2000–2025, tobacco use is still rising in Egypt, registering an overall prevalence of 21.1%, 41.7% among males, and 0.4% among females [14].

Despite the increased incidence of smoking habit in Egypt and the increased risk of developing OMLs and OPMDs, little is known about gender differences regarding the prevalence, and types of oral mucosal lesions, and to the best of our knowledge, no previous studies have compared the prevalence of smoking-related OMLs in males versus females in the Egyptian population. Consequently, this cross-sectional study was designed to assess the prevalence and types of OMLs among male smokers compared to female smokers in an Egyptian population.

## Subjects and methods

### Objective

The present study aimed to assess the prevalence and types of OMLs among male smokers compared to female smokers in an Egyptian population, and investigate whether there was a difference between male and female smokers regarding the prevalence and types of oral lesions.

### Sample size calculation

According to the prevalence of oral mucosal lesions reported recently in Egypt [12], the required sample size was calculated to be 253 for the margin of error or absolute precision of ± 5% in estimating the prevalence with 95% confidence, and was rounded to 300 in each group to improve the power of the study. With this sample size, the anticipated 95% CI is/was (77%, 87%). This sample size was calculated using the Scalex SP calculator.

The current observational hospital-based, two-center cross-sectional study screened in a consecutive way patients who attended the outpatient clinics of the Faculty of Dentistry, The British University in Egypt, and the Faculty of Dentistry, Cairo University.

The study included subjects aged ≥ 20 years with a history of tobacco smoking for at least the last 1 year, forming two groups: 300 male smokers and 300 female smokers.

Along with the patients’ demographic details, information regarding the details of the habit such as duration in years (< 10 years, 11–20 years, 21–30 years, 31–40 years, 41–50 years, > 50 years) and frequency of smoking were recorded using the standardized interviewer-based questionnaire which was developed for this study and an English language version is uploaded as a supplementary file. Smokers were divided into light smokers (smoking 1–10 cigarettes/day), moderate smokers (smoking 11–20 cigarettes/day), and heavy smokers (smoking 20 cigarettes/day) [15].

Exclusion criteria for both groups included patients suffering from systemic disease, e.g., cardiac, respiratory, diabetic, hepatic, gastrointestinal, autoimmune, and renal diseases. Also, all patients under medical treatment, as well as non-Egyptians, were excluded from both groups. In addition, smokeless tobacco, alcohol, and shisha consumers, or other forms of chewed material, were also excluded. Patients who were unwilling to give the complete habit details were excluded. After a detailed explanation of the research scope, patients who agreed to participate in the study signed a written informed consent form prior to the interview and examination.

All participants underwent a thorough case history review and a comprehensive clinical examination.

A thorough clinical examination was conducted for the whole oral cavity. Oral mucosal lesions were diagnosed by evaluating history and clinical examination findings. Regarding leukoplakia and lichen planus, they were diagnosed according to a consensus report of the WHO Collaborating Centre for Oral Cancer [16].

All participants were educated about the hazardous effects of smoking and its adverse effects on both general and oral health. They were all strongly advised to quit this habit.

### Statistical analysis and data interpretation

Data analysis was performed by SPSS software, version 26 (SPSS Inc., PASW Statistics for Windows version 26. Chicago: SPSS Inc.). Qualitative data were described using numbers and percentages. The significance of the results obtained was judged at the 0.05 level.

- Chi-Square and Fisher’s exact tests were used to compare qualitative data between groups as appropriate.
- Binary logistic regression was used to assess the effect of the combination of more than 2 independent variables on dichotomous outcomes using the Stepwise / forward Wald / Enter technique.

## Results

**Fig. 1:**
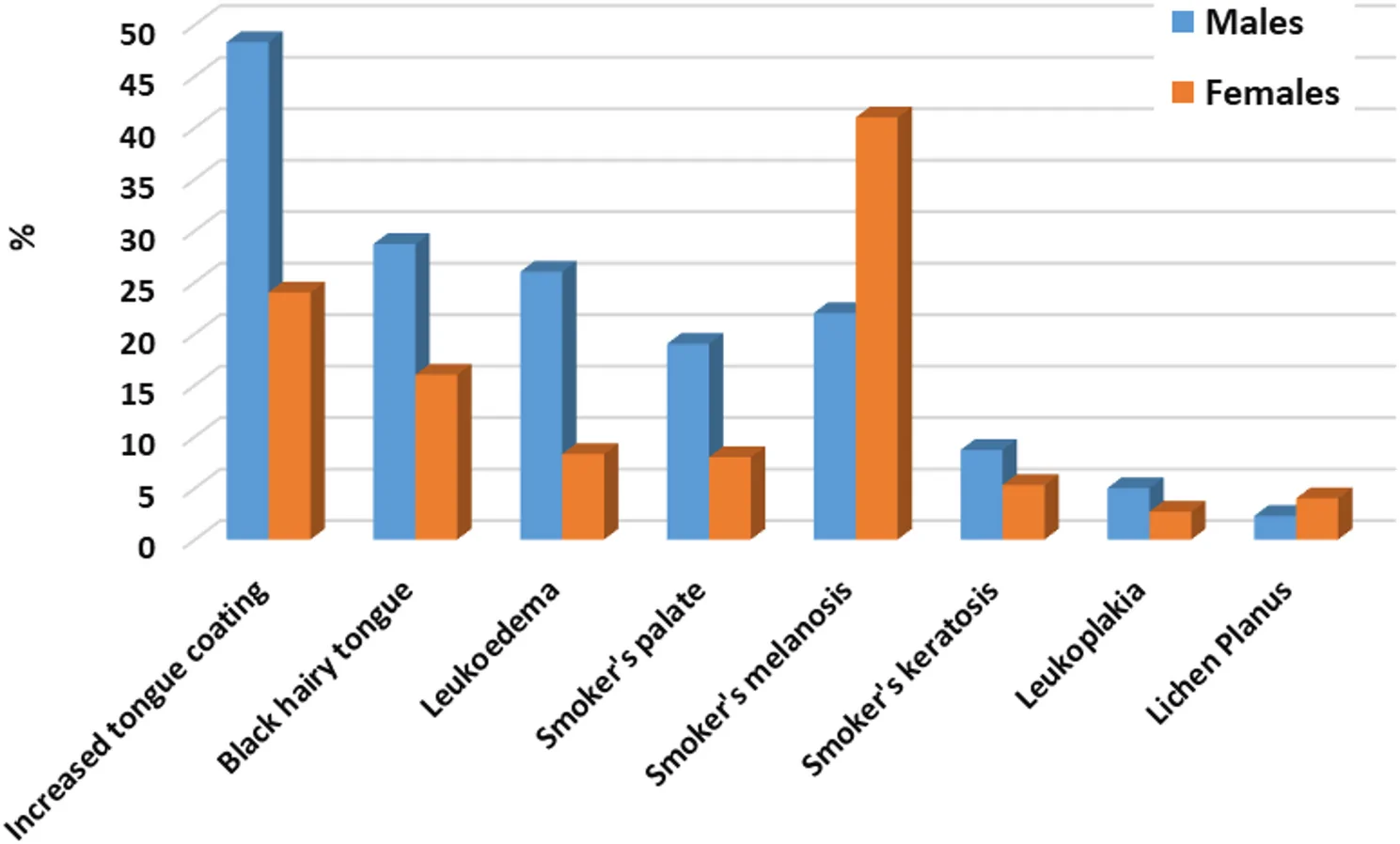
Distribution of oral lesions in males and females

**Fig. 2:**
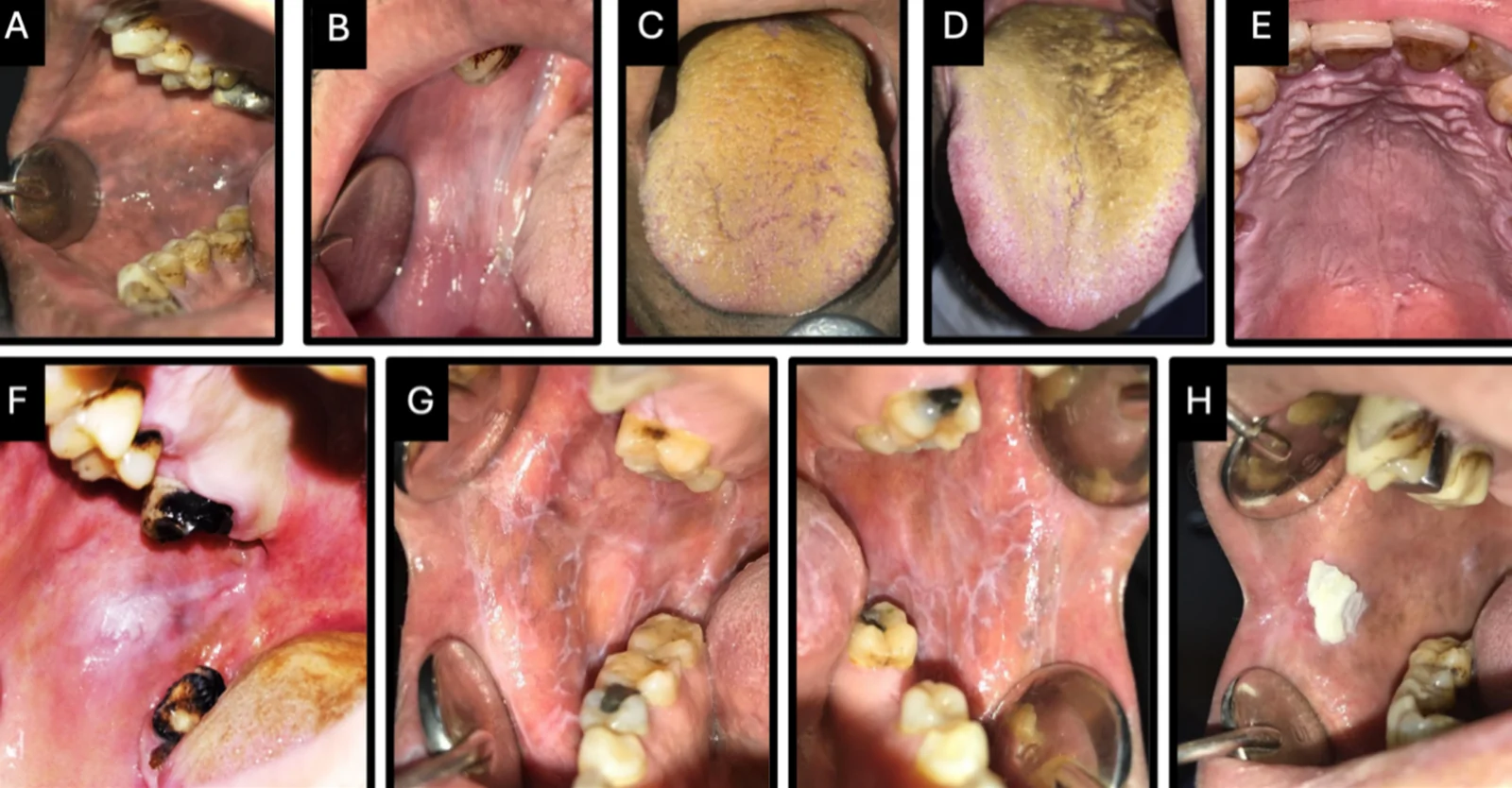
Clinical photographs showing: **A**. Smokers' melanosis. **B**. Leukoedema **C**. Increased tongue coating. **D**. Black hairy tongue. **E**. Smoker's Palate. **F**. Smoker's Keratosis. **G**. Oral Lichen Planus. **H**. Homogenous Leukoplakia

The present study included two groups: males and females, with 300 cases in each. The most frequent age group was 41–50, followed by 31–40 in both groups. As seen in Table 1, the numbers of males and females in each age group match. Table 2 shows the distribution of oral mucosal lesions and their patterns between males and females among the studied cases. Cases reporting Smoker’s melanosis were significantly higher in females, especially on the buccal mucosa and gingiva (Fig. 1). Figure 2 shows examples of various oral mucosal lesions detected. Table 3 shows the odds ratio for different oral mucosal lesions in both males and females. Table 4 demonstrates the predictors of Oral Mucosal Lesions Among Male and Female Smokers. 

**Table 1:** Descriptive statistics of the studied cases, divided into males and females

	Total (N = 600)	Sex			
		Males		Females	
		N = 300	(%)	N = 300	%
Age (years)					
21–30	56	20	6.7	36	12.0
31–40	145	76	25.3	69	23.0
41–50	210	106	35.3	104	34.7
51–60	91	48	16.0	43	14.3
61–70	90	47	15.7	43	14.3
> 70	8	3	1.0	5	1.7
Smoking intensity					
Light	31	10	3.3	21	7.0
Moderate	223	73	24.3	150	50.0
Heavy	346	217	72.3	129	43.0
Duration (years)					
≤ 10	56	21	7.0	35	11.7
11–20	147	79	26.3	68	22.7
21–30	266	133	44.3	133	44.3
31–40	38	20	6.7	18	6.0
41–50	80	42	14.0	38	12.7
> 50	13	5	1.7	8	2.7

**Table 2:** Distribution of oral mucosal lesions and their patterns between males and females among the studied cases

	Males *N* = 300, N (%)	Females *N* = 300, N (%)	Test of significance
Overall prevalence	225(75)	156(52)	ꭓ^2^=34.23 *P* < 0.001*
Increased tongue coating	145(48.3)	72(24)	ꭓ^2^=38.47 *P* < 0.001*
Black hairy tongue	86(28.7)	48(16)	ꭓ^2^=13.88 *P* < 0.001*
Leukoedema	78(26.0)	25(8.3)	ꭓ^2^=32.92 *P* < 0.001*
Smoker’s palate	57(19.0)	24(8.0)	ꭓ^2^=15.54 *P* < 0.001*
Smoker’s melanosis (total)	66(22)	123(41)	ꭓ^2^=25.09 *P* < 0.001*
• Lip	38(12.7)	55(18.3)	ꭓ^2^=3.68 *P* = 0.055
• Buccal mucosa	22(7.3)	55(18.3)	ꭓ^2^=16.23 *P* < 0.001*
• Gingiva	12(4)	41(13.7)	ꭓ^2^=17.41 *P* < 0.001*
Smoker’s keratosis	26(8.7)	16(5.3)	ꭓ^2^=2.56 *P* = 0.110
Lichen Planus	7(2.3)	12(4)	ꭓ^2^=1.36 *P* = 0.244
Leukoplakia (total)	15(5.0)	8(2.7)	ꭓ^2^=2.22 *P* = 0.137
• Speckled leukoplakia	2(0.7)	4(1.3)	FET = 0.673 *P* = 0.686
• Verrucous	2(0.7)	0	FET = 2.01 *P* = 0.499
• buccal mucosa	5(1.7)	5(1.7)	*P* = 1.0
• Gingiva	6(2.0)	1(0.3)	ꭓ^2^=3.61 *P* = 0.057
• Tongue	1(0.3)	1(0.3)	*P* = 1.0
• Palate	3(1.0)	0	FET = 3.02 *P* = 0.249
• Alveolar ridge	7(2.3)	2(0.7)	ꭓ^2^=2.82 *P* = 0.093
• Homogeneous	6(2)	4(1.3)	ꭓ^2^=0.407 *P* = 0.524

ꭓ2 = Chi-Square test, FET: Fisher’s exact test

*statistically significant

#Categories are not mutually exclusive

**Table 3:** Odds ratio for different oral mucosal lesions in both males and females

Oral lesion	Males *N* = 300		Females (R) *N* = 300		*P* value	OR	95%CI of OR	
	-ve	+ve	-ve	+ve			Lower	Upper
Increased tongue coating	155(51.7%)	145(48.3%)	228(76.0%)	72(24.0%)	< 0.001*	4.035 R	2.375	6.854
Black hairy tongue	214(71.3%)	86(28.7%)	252(84.0%)	48(16%)	0.010*	1.942	1.171	3.220
Leukoedema	222(74.0%)	78(26.0%)	275(91.7%)	25(8.3%)	0.001*	2.909 R	1.670	5.067
Smoker’s Palate	243(81.0%)	57(19.0%)	276(92.0%)	24(8.0%)	0.175	1.466 R	0.843	2.549
Smoker’s melanosis	234(78.0%)	66(22.0%)	177(59.0%)	123(41.0%)	0.001* R	.253	0.168	0.383
Smoker’s keratosis	274(91.3%)	26(8.7%)	284(94.7%)	16(5.3%)	0.550	.811 R	0.408	1.612
Lichen planus	293(97.7%)	7(2.3%)	288(96%)	12(4%)	0.100	.423 R	0.152	1.179
Leukoplakia	285(95%)	15(5%)	292(97.3%)	8(2.7%)	0.088	2.687 R	0.863	8.365

*OR *Odds ratio, *r* reference group

**Table 4:** Predictors of the presence of oral mucosal lesions among male and female smokers

Variable	Increased tongue coating			Black hairy tongue			Leukoedema			Smoker's palate			Smoker's melanosis			Smoker's keratosis			Lichen Planus			Leukoplakia:		
	Males	Females	*P* value	Males	Females		Males	Females		Males	Females		Males	Females		Males	Females		Males	Females		Males	Females	
Age (years)																								
21–30	1 (5.0%)	3 (8.3%)	1.0	1 (5.0%)	1 (2.8%)	1.0	4 (20.0%)	0 (0.0%)	0.013*	2 (10.0%)	0 (0.0%)	0.123	4 (20.0%)	9 (25.0%)	0.752	1 (5.0%)	2 (5.6%)	1.0	0 (0.0%)	0 (0.0%)	0.013*	0 (0.0%)	0 (0.0%)	—
31–40	18 (23.7%)	4 (5.8%)	0.003*	5 (6.6%)	6 (8.7%)	0.757	18 (23.7%)	0 (0.0%)	0.001*	18 (23.7%)	3 (4.3%)	0.001*	22 (28.9%)	20 (29.0%)	1.0	9 (11.8%)	0 (0.0%)	0.757	0 (0.0%)	0 (0.0%)	0.001*	0 (0.0%)	0 (0.0%)	—
41–50	44 (41.5%)	9 (8.7%)	0.001/*	17 (16.0%)	7 (6.7%)	0.049*	28 (26.4%)	3 (2.9%)	0.001*	20 (18.9%)	6 (5.8%)	0.006*	21 (19.8%)	39 (37.5%)	0.006*	10 (9.4%)	10 (9.6%)	0.049*	0 (0.0%)	0 (0.0%)	0.001*	0 (0.0%)	1 (1.0%)	0.495
51–60	34 (70.8%)	15 (34.9%)	0.001*	23 (47.9%)	16 (37.2%)	0.397	15 (31.3%)	9 (20.9%)	0.342	6 (12.5%)	6 (14.0%)	1.0	12 (25.0%)	23 (53.5%)	0.009*	5 (10.4%)	2 (4.7%)	0.397	0 (0.0%)	2 (4.7%)	0.342	1 (2.1%)	0 (0.0%)	1.0
61–70	46 (97.9%)	36 (83.7%)	0.025*	38 (80.9%)	14 (32.6%)	0.001*	11 (23.4%)	8 (18.6%)	0.615	11 (23.4%)	9 (20.9%)	0.805	5 (10.6%)	28 (65.1%)	0.001*	1 (2.1%)	2 (4.7%)	0.001*	7 (14.9%)	9 (20.9%)	0.615	11 (23.4%)	4 (9.3%)	0.093
> 70	2 (66.7%)	5 (100.0%)	0.375	2 (66.7%)	4 (80.0%)	1.0	2 (66.7%)	5 (100.0%)	0.375	0 (0.0%)	0 (0.0%)	—	2 (66.7%)	4 (80.0%)	1.0	0 (0.0%)	0 (0.0%)	1.0	0 (0.0%)	1 (20.0%)	0.375	3 (100.0%)	3 (60.0%)	0.464
Smoking																								
Light	5 (50.0%)	0 (0.0%)	0.001*	2 (20.0%)	0 (0.0%)	0.097	0 (0.0%)	0 (0.0%)	—	0 (0.0%)	0 (0.0%)	—	3 (30.0%)	1 (4.8%)	0.087	0 (0.0%)	0 (0.0%)	0.097	0 (0.0%)	0 (0.0%)	—	0 (0.0%)	0 (0.0%)	—
Moderate	16 (21.9%)	5 (3.3%)	0.001*	6 (8.2%)	5 (3.3%)	0.183	3 (4.1%)	0 (0.0%)	0.034*	0 (0.0%)	0 (0.0%)	—	12 (16.4%)	39 (26.0%)	0.128	0 (0.0%)	0 (0.0%)	0.183	0 (0.0%)	0 (0.0%)	0.034*	0 (0.0%)	0 (0.0%)	—
Heavy	124 (57.1%)	67 (51.9%)	0.372	78 (35.9%)	43 (33.3%)	0.643	75 (34.6%)	25 (19.4%)	0.003*	57 (26.3%)	24 (18.6%)	0.116	51 (23.5%)	83 (64.3%)	0.001*	26 (12.0%)	16 (12.4%)	0.643	7 (3.2%)	12 (9.3%)	0.003*	15 (6.9%)	8 (6.2%)	1.0
Duration (years)																								
≤ 10	1 (4.8%)	3 (8.6%)	0.592	1 (4.8%)	0 (0.0%)	0.375	4 (19.0%)	0 (0.0%)	0.016*	2 (9.5%)	0 (0.0%)	0.136	4 (19.0%)	12 (34.3%)	0.360	1 (4.8%)	2 (5.7%)	0.375	0 (0.0%)	0 (0.0%)	0.016*	0 (0.0%)	0 (0.0%)	—
11–20	20 (25.3%)	4 (5.9%)	0.001*	6 (7.6%)	7 (10.3%)	0.577	18 (22.8%)	0 (0.0%)	0.001*	18 (22.8%)	3 (4.4%)	0.002*	23 (29.1%)	17 (25.0%)	0.710	9 (11.4%)	0 (0.0%)	0.577	0 (0.0%)	0 (0.0%)	0.001*	0 (0.0%)	0 (0.0%)	—
21–30	61 (45.9%)	13 (9.8%)	0.001*	29 (21.8%)	14 (10.5%)	0.019*	37 (27.8%)	6 (4.5%)	0.001*	22 (16.5%)	9 (6.8%)	0.021*	27 (20.3%)	52 (39.1%)	0.001*	12 (9.0%)	10 (7.5%)	0.019*	0 (0.0%)	2 (1.5%)	0.001*	1 (0.8%)	1 (0.8%)	1.0
31–40	17 (85.0%)	12 (66.7%)	0.260	11 (55.0%)	9 (50.0%)	1.0	7 (35.0%)	6 (33.3%)	1.0	4 (20.0%)	6 (33.3%)	0.468	5 (25.0%)	11 (61.1%)	0.047*	3 (15.0%)	2 (11.1%)	1.0	1 (5.0%)	0 (0.0%)	1.0	0 (0.0%)	0 (0.0%)	—
41–50	42 (100.0%)	34 (89.5%)	0.047*	35 (83.3%)	13 (34.2%)	0.001*	10 (23.8%)	7 (18.4%)	0.596	10 (23.8%)	5 (13.2%)	0.262	5 (11.9%)	25 (65.8%)	0.001*	1 (2.4%)	2 (5.3%)	0.001*	6 (14.3%)	8 (21.1%)	0.596	10 (23.8%)	3 (7.9%)	0.071
> 50	4 (80.0%)	6 (75.0%)	1.0	4 (80.0%)	5 (62.5%)	1.0	2 (40.0%)	6 (75.0%)	0.293	1 (20.0%)	1 (12.5%)	1.0	2 (40.0%)	6 (75.0%)	0.293	0 (0.0%)	0 (0.0%)	1.0	0 (0.0%)	2 (25.0%)	0.293	4 (80.0%)	4 (50.0%)	0.565

Used test: Fisher’s exact test, Chi-Square test

*statistically significant, data expressed as number (%)

## Discussion

Smoking represents not only a chief health hazard, but it is also a major risk factor for developing several oral mucosal lesions. Such lesions can include potentially malignant disorders with or without epithelial dysplasia and a subsequent increase in the risk for the development of oral cancer [17]. The WHO states that nearly 6 million deaths occur every year as a result of tobacco use, which may escalate to 8 million deaths a year by 2030 [2].

Historically, smoking rates have been higher in males than in females, leading to a greater cumulative exposure to the harmful chemicals in tobacco [18]. It has also been claimed that men tend to smoke more heavily (i.e., more cigarettes per day) and for longer durations, which increases their risk of developing oral mucosal lesions. Many other postulations were presented, such as that developed countries have a much higher smoking prevalence among females than developing countries [18]. In Egypt, smoking prevalence among males was previously reported to be significantly higher than in females [4, 19]. However, an increase in smoking rates has been recently reported among Arab females, in general, including Egyptians [19]. The descriptive data presented in the present study can be considered evidence that not only has an increase in the number of smoking females taken place, but also there is a change in the pattern and overall habits related to smoking in Egyptian females. The male and female groups in the current work share similar features of age distribution, duration, and intensity (frequency) of smoking.

To the best of our knowledge, this study is the first to investigate gender differences concerning the prevalence of smoking-related OMLs in Egypt. All prevailing studies are either collecting smokers, whether males or females, within the same group, or assessing the prevalence of oral lesions among male smokers only [12].

The overall prevalence of OMLs (in both males and females combined) in the present results is 63.5%, aligning with a previous study in South India that reported an OML prevalence rate of 64% [20], as well as other studies that reported a prevalence of 61.8% among different populations [21, 22]. However, the current prevalence of OMLs was higher than in some previous studies (28%, 26.75%) [1, 23]. These differences between studies may be due to the lack of standardized methods, such as variations in age ranges, study population sizes, criteria for identifying oral lesions, and types of oral mucosal lesions. Many factors can influence the results, including the mean age of the sample; for example, older populations tend to have a higher percentage of oral mucosal lesions than younger ones [4]. Fortunately, the present study included patients across various age groups, from 20 to 70 years and older. Nevertheless, most cases in both groups were within the fourth and fifth decades, where males and females, treated as separate groups, appeared to be age-matched.

The types of lesions revealed by the present study were increased tongue coating, black hairy tongue, leukoedema, smoker’s palate, smoker’s keratosis, lichen planus, and leukoplakia. No cases of erythroplakia or squamous cell carcinoma were found, in contrast to previously reported results [9, 10].

In the present study, when the two groups were compared, the prevalence of OMLs was higher in males than in females, despite the aforementioned similarity in descriptive data between them. This is consistent with several previous studies among different populations [4, 22, 24, 25]. The current results revealed that 75% of males presented with one or more oral lesions which was significantly higher than females (52%), this is in line with a previous research showing a greater ratio of OMLs among males (73%) versus females (27%), however, this study included a higher number of the males versus female patients [25], whereas the number of patients in our study was equal in both groups.

On the other hand, another study showed that oral lesions affected 64.5% of males and 59.8% of females but did not demonstrate a statistically significant difference [22]. Similarly, the prevalence of OMLs was 46.3% in males and 41.3% in females, with no significant difference in another study [24].

When individual lesion types were considered, increased tongue coating, black hairy tongue, leukoedema, and smoker’s palate were found in males more than in females, with a statistically significant difference.

In general, cigarette smoke affects the oral mucosa and the tongue’s bacterial community and morphology and thus may contribute to the higher prevalence of coated tongue in smokers, which can lead to oral malodor and black hairy tongue [26]. In the current study, increased tongue coating was the most common oral lesion, which is in line with prior studies [22, 27], and it was significantly more reported in males, as they were found to be at higher risk of having increased tongue coating than females, with an odds ratio (OR = 4.035). The difference was of high statistical significance among ages ranging from 31 to 70 years, among light and moderate smokers, and within the smoking duration of less than 50 years. However, with heavy smokers and smoking duration of more than 50 years, the difference gap nearly disappeared.

Males were also found to be at a higher risk of developing black hairy tongue than females (OR = 1.942). It registered the highest statistical significance among age groups 41–50 and 61–70 years, and when the smoking duration was 21–30 and 41–50 years, whatever the smoking intensity.

Among the lesions detected in the present study is smoker’s palate, which is a condition in tobacco smokers that is associated with a visible whitish lesion and opaque appearance in the oral mucosa due to the thickening of the keratin layer with inflammation of the minor salivary ducts of the palate. This occurs due to the heat generated by smoking cigarettes and bidis, not as a result of the chemicals in the smoke. It can increase the risk of oral squamous cell carcinoma in certain areas [21, 28]. In the current study, it was found only among heavy smokers of both genders, and more in males than in females. Its increased prevalence among males is in agreement with previous research [20, 21, 29]. With an odds ratio of 1.466, males seemed to be at a higher risk of developing the lesion. The highest statistically significant difference was among the age groups 31–50 years, smoking duration 11–30 years. With a longer duration of smoking, the highly significant difference disappears. Thus, it seems that females need a longer period to develop the lesion. Actually, females are reported to care more about oral hygiene than males [30, 31]. This can have a direct effect on the clearance of tobacco chemical combustion products, thus diminishing their impact on the palatine tissues and related minor salivary glands.

Leukoedema, a whitish oral mucosa lesion, is common among smokers and can result from persistent irritation and trauma. This condition has been neglected for too long, and if left untreated, it may increase the risk of cancer [32]. Males are at a higher risk of developing leukoedema than females, with an odds ratio of (OR = 2.909) according to the present results. This agrees with earlier research reporting a higher occurrence of leukoedema in males [20]. It registered the highest statistical difference among the age groups 21–50 years and with smoking duration from ≤ 10 up to 30 years. Thus, again, females need more time to develop leukoedema than men. They also seem to develop it only with heavy smoking. This could also be related to different levels of oral hygiene between genders [30, 31].

Smoker’s keratosis was also more commonly encountered in male smokers in the present results, and this again agrees with previous studies [29].

Contrarily, smokers’ melanosis was higher in females, especially those located on the buccal mucosa and gingiva. The highly significant difference was evident in the age groups 41–70 (41–50, 51–60, 61–70), among heavy smokers in particular, and when the duration of smoking ranged from 21 to 50 years. Smokers’ melanosis is a common brown pigmentation of the oral mucosa in smokers caused by the chemicals contained in cigarettes. It affects 21.5–30% of smokers, mostly women [22], which agrees with the present results showing that the prevalence of smoker’s melanosis was higher in females, especially those located on the buccal mucosa and gingiva, and that males are at lower risk of developing smoker’s melanosis (OR = 0.253) than females. This also agrees with previous research indicating that smoking exerts a significant and detrimental impact on gingival melanosis [33, 34]. The higher prevalence of smokers’ melanosis in females could be attributed to the increased estrogen levels in various life periods. The effect is obvious in the younger age group, where estrogen levels and the endocrine impact could still be appreciable, then as women become older, the melanin-enhancing aging process probably continues what estrogen has previously started, as they begin from an already exaggerated response [35, 36].

Our results also showed that the age group 51–60 in both males and females was at a much higher risk of developing smoker’s melanosis (OR = 12.712) and heavy smokers were at a much higher risk of developing smoker’s melanosis (OR = 6.961), which is in line with other studies reporting that the number of cigarettes smoked per day is directly proportional to gingival pigmentation, with a marked increase in prevalence for individuals who smoke more than 10 cigarettes daily [34].

In the present study, leukoplakia, a frequently observed potentially malignant disorder linked to tobacco use, was more common in males than females, aligning with earlier studies [20, 29, 37]. Although the difference in leukoplakia prevalence was not statistically significant, the odds ratio indicated more than twice the risk for males compared to females (OR = 2.687). Another potentially malignant disorder with a 10.19% rate of dysplasia [38, 39] is lichen planus, which was more frequently found in females (4%) than males (2.3%) in this study. This is consistent with the higher incidence of the condition in females compared to males [4], which exceeds the impact of smoking.

Several factors could lead to the increased prevalence of smoking-related OMLs in males, and even some of them in particular. Males might have a less robust immune response in the oral cavity, as immune surveillance by antigen-presenting and processing cells are decreased, leading to increased inflammation and a higher likelihood of lesion formation [40]. Furthermore, males have higher levels of androgens (e.g., testosterone), which might make their oral tissues more susceptible to the carcinogenic and inflammatory effects of smoking. Actually, smoking increases androgen pathway signaling, and androgens, in turn, increase the expression and activity of TMPRSS2 alongside ACE2 upregulation in the oral epithelium, leading to more severe tissue response [38]. Androgens have been linked to the increased formation of reactive oxygen species and higher rates of cellular turnover, which may increase vulnerability to tobacco-related damage. Lu et al. 2010 reported that androgens increase the basal levels of ROS. When ROS generation is increased, it disrupts redox homeostasis and induces oxidative stress conditions. ROS impairs the functionality of critical biomolecules and organelles like DNA, proteins, and lipids, leading to various diseases [41]. In contrast, estrogen, which is more prevalent in females, may have protective effects on the oral mucosa. Some studies suggest that estrogen may help modulate the inflammatory response to injury and reduce the severity of certain types of mucosal lesions [42].

Moreover, it was found that the levels of gene expression differed in the oral mucosa of males vs. females in response to smoking [43]. Such gender-dependent differences in smoking-induced gene expression, with the resultant differences in oral transcriptomics in smokers, could furnish some explanation for the significant differences in the prevailing OMLs in either category.

Also, Women are generally more likely to visit healthcare providers for routine check-ups and health concerns, which may result in earlier detection of oral lesions in females. On the other hand, men may be less likely to seek care for minor symptoms, which could contribute to the perception that lesions are more common in males, especially when they are left untreated. Females were reported to be screened for oral cancer more than males, despite that males are diagnosed with oral cancer at a male-to-female ratio of 2:1 [44, 45].

When examining predictors of oral lesions among males, a statistically significant increase in prevalence was found to be associated with increased tongue coating, black hairy tongue, and lichen planus in the 61–70 age group. Conversely, leukoplakia was significantly more common in individuals above 70 years. This aligns with previous studies indicating that the overall prevalence of OMLs is higher in older individuals than in younger ones and that oral lesions are more frequently observed in those over 60 years [20, 46].

Additionally, the occurrence of increased tongue coating, black hairy tongue, leukoedema, smoker’s palate, smoker’s keratosis, and leukoplakia was significantly higher with heavy smoking in males. Increased tongue coating, black hairy tongue, and lichen planus were notably more common with a 31–40 year smoking duration, and leukoplakia was significantly more prevalent with a 41–50 year smoking duration.

When the predictors of oral lesions among females were studied, a statistically significant increase in prevalence was revealed for the smoker’s palate among the 61–70 age group. Moreover, the prevalence of increased tongue coating, black hairy tongue, leukoedema, smoker’s melanosis, and leukoplakia was significantly increased in the age group above 70 years. The prevalence of lichen planus was significantly increased in both age groups. These observations are in accordance with previous reports. Oral lesions were more common among patients aged 50 years or older (37.8%) than among those aged 20 to 35 years (32.4%) [25]. Another research showed that OMLs were most commonly found to affect the age group of 56–65 years [4], or even older age groups. Actually, adults aged 65–74 years were found to have the highest prevalence of oral mucosal lesions in a study performed in India [47].

When the duration of smoking was considered, the prevalence of increased tongue coating was significantly increased with a 31–40 year duration of smoking. On the other hand, black hairy tongue, leukoedema, smoker’s melanosis, leukoplakia, and lichen planus were significantly increased with a 41–50 year duration. Thus, a history of longer duration of tobacco usage could be the reason for the occurrence of lesions among older age group patients, which is in accordance with prior research [33].

In addition, a statistically significant increase in the prevalence of all lesions was found with heavy smoking in females. It has been previously stated that smokers presented with different types of individual and combined lesions that were strongly associated with the frequency and duration of tobacco usage [44].

This study acknowledges a few limitations, including a cross-sectional design, which hinders causal inference and the ability to track changes in tobacco usage and oral health over time. Moreover, reliance on self-reported data may introduce potential information bias. Furthermore, smoking is not quite tolerable in Egypt, especially among women. As a result, respondents, particularly female participants, may have answered according to these social norms. However, self-reported smoking behaviors are a valid method to measure smoking consumption [18]. In addition, as the findings were derived from patients attending the outpatient clinics of two hospitals, therefore, the findings from this study may not be generalized to the entire Egyptian population.

Regarding the strengths of this study, they include the large sample size, age matching, and an equal number of cases in gender groups, which allow for accurate comparison of prevalence and correlation of oral lesions and various variables between males and females. Additionally, the study involved comprehensive clinical assessments of the oral mucosa for the precise diagnosis of OML. The cases diagnosed with leukoplakia and lichenoid lesions will be monitored with periodic clinical examinations to detect any possible malignant transformation in those prevalent OPMDs.

The agreement of the present results with other previous research that was carried out among other populations, which differ in culture and ethnic background, confirms the increased vulnerability of males to smoking-related OMLs. If we add the fact that males exhibit substantially higher odds of having OPMDs compared to females (OR = 8.1, *p* < 0.01) [37] and the fact that tobacco smoking increases the number of aneuploid nuclei in the oral epithelium and causes oral malignant and premalignant lesions [21, 48], together with the lack of regular check-up among males, this should ring an alarm bell to the multitude of oral health risk hazard among male smokers.

## Conclusion

Within the limitations of the present study, it could be concluded that both males and females manifest the same types of oral mucosal lesions in relation to smoking; however, males have an overall significantly higher prevalence of these lesions. This emphasizes the need for gender-specific strategies for oral health interventions and awareness campaigns. A crucial role should be carried out by dentists and other healthcare providers regarding targeted male smokers’ education and clinical oral screening.

### Recommendation

Further studies, including comparisons of demographic data and dose-response relationships with oral lesions with larger community-based studies, should be conducted to estimate more representative percentages of oral lesions. Studies observing the effects of smoking cessation on oral health are also recommended to provide evidence regarding the chances of reversal of oral mucosal lesions, which can be a great tool for patients’ education.

## Supplementary Information

Supplementary Material 1.

## Data Availability

All data generated or analyzed during this study are included in this published article.
