# Supplementary material for: Prevalence of oral mucosal lesions among smokers based on gender: a bicentric cross-sectional study in Egypt
Source: BMC Oral Health. 2026 Nov 3;26:2171. doi: 10.1186/s12903-026-10040-y (PMC13729621; doi:10.1186/s12903-026-10040-y)
Supplement: Supplementary file 1 [file 12903_2026_10040_MOESM1_ESM.docx]

**Prevalence of Oral Mucosal Lesions among Smokers** **Based on Gender: A Bicentric Cross-Sectional Study in** **Egypt**

**The study questionnaire:**

| 1. **Do you agree to participate in this study?** | A) Yes B) No |
| --- | --- |
| 2. **Name** |  |
| 3. **Age** | A. 21-30 years B. 31-40 years C. 41-50 years D. 51-60 years E. 61-70 years F. >70 years |
| 4. **Sex** | A. Male B. Female |
| 5. **Smoking intensity:** | A. Light smokers (1-10 cigarettes/day) B. Moderate smokers (11-20 cigarettes/day) C. Heavy smokers (≥20 cigarettes/day) |
| 6. **Smoking duration:** | G. ≤10 years H. 11-20 years I. 21-30 years J. 31-40 years K. 41-50 years L. >50 years |
| **Clinical Examination:** |  |
| **1. Increased tongue coating** | A. Yes B. No |
| **2. Blach hairy tongue** | A. Yes B. No |
| **3. Leukoedema** | A. Yes B. No |
| **4. Smoker's palate** | A. Yes B. No |
| **5. Smoker's melanosis** | A. Lip B. Buccal mucosa C. Gingiva D. Alveolar ridge E. Palate F. Others G. No |
| **6. Smoker's keratosis** | A. Lip B. Buccal mucosa C. Gingiva D. Alveolar ridge E. Palate F. Others G. No |
| 7. **Lichen Planus** | A. Yes B. No |
| 8. **Leukoplakia** | A. Lip B. Tongue C. Buccal mucosa D. Gingiva E. Alveolar ridge F. Palate G. Others H. No |

**Leukoplakia type:**	A. Yes

B. No
